# “You Can’t Meet Everyone's Needs After-Hours”: After-Hours Domestic and Family Violence Services in Rural and Remote Areas

**DOI:** 10.1177/10778012231183655

**Published:** 2023-07-02

**Authors:** Natalia Hanley, Catherine MacPhail

**Affiliations:** 1 School of Health and Society, 224518 University of Wollongong, Wollongong, NSW, Australia

**Keywords:** domestic and family violence, after-hours services, rural and remote Australia, victim–survivor

## Abstract

Domestic and family violence is a significant issue in the Murrumbidgee region of New South Wales, Australia, mirroring national and international concerns about gender-based violence. Generally, there are known barriers associated with providing domestic and family violence (DFV) services in rural and remote communities; however, little research has considered the specific service needs and service barriers in the after-hours period. This is crucial. The already limited rural and remote services available during business hours are further constricted in the after-hours period. This article reports on research about after-hours service need and service challenges in six target communities in the Murrumbidgee region.

## Introduction

There is a growing body of literature that has detailed high rates of domestic and family violence^1^ (DFV) and service gaps and barriers in regional, rural, and remote communities in Australia (Dillon et al., 2015; Hooker et al., 2019; Meyer & Stambe, 2021; Ragusa, 2013; Ragusa, 2017; Wendt & Hornosty, 2010). The most recent Australian National Community Attitudes towards Violence against Women Survey (NCAS) report (Webster et al., 2018) suggests that women residing in rural and regional areas^2^ may experience DFV that is more severe and more prolonged than women in other areas. In the Western region of New South Wales (NSW), Australia, the Murrumbidgee Primary Health Network (MPHN) supports primary healthcare services and is tasked with improving health outcomes (MPHN, 2021). As part of this task, MPHN commissioned this research on after-hours DFV service needs in the region. The area serviced by MPHN is extremely large, covering 124,413 km^2^, contains 21 local government areas (LGAs), and has almost 250,000 residents spread across 514 communities (MPHN, 2021). The large area, which includes regional, rural, and remote communities, presents challenges for service delivery in many sectors, including DFV, which typically occurs repeatedly in the after-hours period, and may commonly require a rapid response (Owen & Carrington, 2015).

Specific concerns about rates of DFV in the Murrumbidgee region that gave rise to this research are borne out in data from the NSW Bureau of Crime Statistics and Research (BOSCAR). Overall, many parts of the region report higher rates of DFV than the NSW state average. In the Narrandera LGA, for example, the rate of DFV assault was 932.4 incidents per 100,000 population, against a NSW state average of 394.9 from April 2020 to March 2021 (BOSCAR, 2021). Despite high levels of DFV, attitudinal data from a community-based survey in Wagga Wagga (one of the largest towns in the Murrumbidgee region), based on the national NCAS survey, found that local residents were able to identify a range of behaviors that constitute DFV, though with less recognition of nonphysical forms of DFV. Participants acknowledged that DFV was a common and serious issue in their community and that it is gendered, with men more likely to perpetrate DFV. However, around 4% of participants held attitudes that justified DFV, and more than one third of the participants believed that women report made up DFV incidents (Schineau & Darley-Bentley, 2016).

Against this backdrop, this research sought to understand DFV service needs and challenges in six target communities in the Murrumbidgee region. The communities were identified by MPHN and all had higher than state average rates of DFV. Our research had three broad aims: (a) to understand the experience of DFV in target communities in the Murrumbidgee region, (b) to identify appropriate models of care to underpin DFV services for women and families, and (c) to understand DFV service needs in the after-hours period. This article specifically reports on the third aim, the provision of after-hours services in this rural health district. This is an important aim for several reasons; there is some (though dated) international research that points to the majority of DFV occurring between 6 p.m. and 6 a.m. (Klein, 2009). There are reduced avenues available to access support in this period, and there is a paucity of research literature exploring the need for and availability of support in the after-hours period. Where available literature exists, the focus is on crisis accommodation, which is an important but narrow framing of multifaceted after-hours needs.

### DFV After-Hours Services

In the provision of DFV services, after-hours refers to any period outside of traditional business hours (9 a.m. to 5 p.m. Monday to Friday), though some services' normal operating hours may extend beyond these hours. After-hours also includes public holidays (Family Safety Victoria, 2017). Four main service domains are discussed in the after-hours DFV literature: policing, emergency medical services, telephone helplines, and crisis accommodation. Most of the existing literature is focused on policing and crisis accommodation and typically focuses on issues such as barriers to access and service quality.

#### Policing DFV

Police are often the first responders to DFV that occurs in the after-hours period. However, some rural police stations close during the after-hours period, with calls referred through to larger stations that may be some distance away. General duties officers are most often the first responders to DFV whether after-hours or not.

The literature identifies specific groups of individuals who might commonly experience barriers to accessing policing services, including Indigenous people and people living in rural areas. Indigenous victim–survivors experience unique barriers in reporting DFV, including discriminatory practices and a lack of cultural safety when in contact with police (Douglas & Fitzgerald, 2018; Fiolet et al., 2021; Funston, 2013; Meyer & Stambe, 2021). One outcome, according to Blagg et al. (2015) is that underreporting of DFV is likely higher among Indigenous women than non-Indigenous women, though the authors acknowledge the lack of empirical evidence for this assertion. Additional barriers in a rural setting are as follows: close relationships between local police and perpetrators, confidentiality issues, and police in these communities sometimes relying on their own discretion, with inequitable outcomes. Conversely, quantitative literature suggests that as a broad group of people, victim–survivors in rural communities are more, or just as likely, to report DFV to police due to the poor availability of alternative options for assistance (Dowling et al., 2018, p. 24).

#### Emergency Health Services

Emergency health services are also commonly the first or second point of service contact for victim–survivors of DFV, particularly in the after-hours period. In Australia, emergency departments have been identified as the third most utilized option after friends/family and police for people seeking help because of DFV (Australian Institute of Health and Welfare [AIHW], 2018; Olive, 2007; Reisenhofer & Seibold, 2007) and screening for DFV is routinely undertaken (Spangaro et al., 2010, 2011). The latest, now dated, edition of NSW Health Policy and Procedures for identifying and responding to domestic violence was republished in 2006, in relation to after-hours services it states that:

Women often present to Emergency Departments between the hours of 5 p.m. and 8 a.m., when few or no social work services are available. This fact, coupled with the low identification and the prevalence of domestic violence indicate the need for training on this issue for doctors and nurses in Emergency Departments and for the provision of appropriate backup referral services such as after-hours social work services. (NSW Health, 2006, p. 35)

In comparison, during 2007 between 31% and 54% of female patients in Australia, the United Kingdom and the United States accessed an emergency department as a result of experiencing DFV (Reisenhofer & Seibold, 2007, p. 7). Access to services beyond initial DFV screening in health settings such as emergency departments is varied and particularly dependent on location, with two thirds of women in metropolitan areas accessing a social worker, compared to only a third and less than a quarter in two rural sites (Spangaro et al., 2020).

#### Technology-Based Services

A major after-hours option for victim–survivors is technology-based services, usually accessed through mobile telephones. Technology has opened space to explore self-help and e-technology support on a wide range of issues including DFV (Wendt et al., 2015). Technology-based services are being used increasingly in Australia where there are large and spread-out communities and geographical barriers to accessing assistance. Technology-based services take multiple forms including telephone advice and referral, email counseling, chat rooms, and videoconferencing.

#### Crisis Accommodation

The most reported need for victim–survivors is immediate and safe accommodation. Consequently, a large volume of the existing literature focuses on this issue (e.g., see Blagg et al., 2015; Wendt et al., 2015). Wendt et al. (2015) outline the importance of crisis accommodation for the early response to DFV. Crisis accommodation affords women and children time to seek longer term housing, though there is concern around privacy and safety of service users and staff in regional, rural, and remote communities. In such areas, “close community ties and a lack of anonymity, [mean that] perpetrators often know which homes are the designated safe homes” (Wendt et al., 2015, p. 22), and this necessitates more creative and flexible crisis accommodation solutions.

The literature notes that to ensure women's safety, crisis accommodation may not accept women and children who live in the region where the crisis accommodation is located, thus requiring women and families to relocate away from their support networks to places where they have no contacts or existing relationships. This is identified as a particular concern for Indigenous women due to their connectedness to families and communities. As a consequence, women can feel powerless because of the curtailed options available to them (Blagg et al., 2015).

### DFV Service Structures in Regional, Rural, and Remote Communities

A common service structure is the hub and spoke model that is discussed in the regional, rural, and remote context by Wendt et al. (2015). In this model, a central hub is located in a regional center and provides outreach to remote populations. This can be highly effective when frontline staff know and consult with local people and there is sufficient staffing and resourcing. Hub and spoke models improve access and use through attachment to local, community-owned infrastructures that increase the credibility and relevance of specialist services. There is potential for the hub and spoke model to strengthen service coordination and thereby be particularly useful for visiting specialist service integration into community (Wendt et al., 2015). That said, the hub and spoke model is also contested, particularly regarding its implications for Indigenous service users because the result of this model has commonly been a withdrawal of services from rural and remote areas (Prout & Centre for Aboriginal Economic Policy Research, 2008).

In Australia, DFV services, including after-hours services, are delivered from varying settings including single communal shelters, multibuilding cluster models of self-contained units with counseling rooms, children's resource buildings, playgrounds, and group or training rooms and shop fronts. In remote areas, there may be purpose-built facilities or a single/small number of outreach workers working from other organizations’ spaces. This means that there are community development workers, counselors, domestic violence liaison officers, social workers, service coordinators and referral operators, children's workers, and outreach workers in isolated communities often with little support. These services may specialize in certain disadvantaged groups of women and children in their community (Oberin, 2018).

There are three main entry points to the range of services identified: (a) justice and statutory services that include police, family courts, and child protection services; (b) mainstream nonspecialist services including health and education services; and (c) specialist domestic family violence and/or sexual assault services that include crisis accommodation and other housing and homelessness services, crisis services, forensic services, counseling, financial assistance, and perpetrator intervention services (AIHW, 2018).

### Barriers to DFV Services in Regional, Rural, and Remote Communities

There are four interconnected barriers to access identified in the literature: transport, privacy and confidentiality, shame and stigma, and lack of culturally appropriate services. The literature acknowledges some of these barriers across geographic spaces in Australia and internationally. For example, in their systematic review of international literature, Robinson and Spilsbury (2008) note that lack of support for cultural and linguistically diverse survivors, guilt and shame, and security and confidentiality concerns act as barriers to seeking support from health services. However, these recognized barriers are magnified and compounded in the regional context (see Wrathall & Herron, 2021 for a Canadian example).

The distance between major service centers and where victim–survivors live makes it challenging for people to attend services, particularly as transport options are reduced in regional compared to metropolitan settings (Owen & Carrington, 2015). Apart from physical access to services, in nonurban areas, victim–survivors have reported concern around privacy given that in small communities, people are more known to each other (Campo & Tayton, 2015). Consequently, Wendt et al. (2015) describe how victim–survivors of DFV in regional, rural, and remote areas rely on informal care and support networks to a greater extent than in urban areas. Indigenous and culturally and linguistically diverse women face several specific barriers to accessing appropriate services, both generally, and more specifically in nonmetropolitan areas (Maher & Segrave, 2018; Murray et al., 2019). Recent Canadian health research identified the layers of inequity, or “double burden” of access to healthcare services that exist for people in rural areas, and especially immigrants settling in rural communities (Patel et al., 2019, p. 678). These barriers are cultural and linguistic, and among Indigenous women, there is also a high degree of mistrust based on previous experiences with welfare and legal systems (WESNET, 2000). The service barriers First Nations survivors experience are also described in international literature. For example, Rizkalla et al. (2020) highlight fragmented services, lack of cultural safety, and the complexity of privacy and confidentiality in small communities as just some of the barriers. The success of responses to DFV for Indigenous survivors relies on understanding the context of colonization, disadvantage, oppression, and marginalization (Blagg et al., 2015).

## Methodology

A mixed methods approach was used to collect relevant information about access to, and need for, after-hours DFV services in the target communities. The mixed methods design enabled consideration of different stakeholder perspectives and “allows respective strengths and weaknesses of each approach to complement each other” (Regnault et al., 2018, p. 2). First, we conducted a rapid review of the literature to ascertain what is known about after-hours DFV services and what services are provided in the region. Second, we interviewed service providers, including managers and frontline service providers. Third, we carried out four community fora across the study sites and, fourth, community observations in some target communities.^3^ A project steering committee^4^ provided advice and guidance via telephone, video call, and email. In line with the focus of this article, we have drawn on the interview and community fora data in the Results and Discussion sections.

### Interviews With Managers and Frontline Service Providers

Following Wrathall and Herron, we recognize that service providers are “… key informants who engage with a broad range of rural service users because, as frontline workers, they have knowledge into the experiences of those who are victim–survivors of IPV, those who perpetrate IPV, and the community dynamics that surround these experiences” (2021, p. 3). We identified DFV service provider managers and workers^5^ through the steering committee, web-based service searches, and, thereafter, snowball sampling. We conducted interviews with managers and frontline providers of services that included nongovernment support services for women survivors, the police, and government support services (*n* = 8 interviews, including 11 participants). Several individuals that we approached declined to be interviewed or failed to finalize an interview time after initially agreeing to participate (*n* = 16), despite follow-up. We believe that resource constraints made it difficult for some staff to prioritize research participation with significant competing demands.

The interviews took between 45 and 60 min. Prior to engaging in the interview, participants were provided with a participant information sheet and consent form. We developed individual interview topic guides for managers and frontline service providers. The topic guides initially sought background information about the service that the participant represented and their role in it, followed by eliciting information about the women survivors of DFV making use of their service. The interview then focused specifically on the Murrumbidgee region and included questions such as “What sort of DFV support and/or services have good coverage in this area?” and “What are the major after-hours service needs for women who are experiencing DFV and their families?”. We further explored the after-hours component through questions such as “Thinking specifically about after-hours services, what would typically be available to a woman who needs the following services on a Saturday night?”. We provided participants with several DFV management issues such as emergency accommodation, making a police report, financial support, etc., and repeated the question with a focus on other after-hours time periods. Finally, we asked what would enable their own service to offer after-hours service to survivors of DFV. The majority of interviews were conducted over the phone.

### Community Fora

Posters advertising the community fora were circulated to steering committee members and to local organizations in the target communities for distribution. In addition, posters were uploaded to online social media platforms in these communities. Community fora were conducted in local venues in the target communities. At all community fora, we made provision for service providers to be available to provide support to participants who might become distressed by the discussion of DFV, despite the parameters of the discussion being about service provision and not personal experiences. The community fora provided an informal setting in which participants were encouraged to raise issues relating to accessing DFV services and assistance during the after-hours time period. Four community fora were conducted with a total of 15 participants (ranging from 2 to 5 participants each). The community fora were intended to target general community members from the four identified towns; however, participants tended to be people working in DFV services, as well as being local community members, and participation was less than hoped for.

### Data Analysis

Recordings from interviews and community fora were professionally transcribed and imported into Nvivo (version 11) for analysis. Following a framework analysis approach (Pope et al., 2000), we created a code frame based on *a priori* knowledge of DFV after-hours service provision issues and the topic guide developed for the study. Coding was conducted by a single researcher using this code frame while also allowing for new emerging themes and ideas to be inductively developed through adding new codes during the coding process. Analysis was performed through a process of initially reading and rereading the transcripts, coding according to the coding frame, and emerging ideas and repeated discussion and refining of the major themes among the authors. Themes and subthemes were established based on a process of constant comparison (Corbin & Strauss, 2008). Themes and subthemes were further explored by extracting illustrative quotes from the coded data if they reflected dominant attitudes or illustrated counter normative ideas or concepts. Framework analysis was used as it provides a structured approach to analysis but with sufficient flexibility to remain iterative in consideration of the data. The systematic nature of the approach allows for a clear audit trail as the coding progresses and encourages thinking about the depth of themes (thematic analysis) as well as comparison through case analysis of different participants through the use of framework matrices in Nvivo (Hackett & Strickland, 2018).

Data accuracy and saturation were checked throughout the analysis process to ensure credibility, while we attempted to ensure transferability through recruitment of a range of stakeholders across service provision types and communities in the region. Interpretation of the data was assisted through repeated and in-depth discussion of the data among the research team and therefore ensures dependability. Together with the ongoing reflexivity to achieve confirmability, these processes ensured the trustworthiness of the data analysis (Lincoln & Guba 1985).

## Results

Throughout the discussions across both interviews and community fora, there was an acknowledgement that DFV victim–survivors, particularly in rural communities, have complex needs that are challenging to meet, even within usual business hours. Participants acknowledged that the after-hours period was particularly difficult for survivors, mostly as a consequence of unavailable or reduced service hours or reduced transport options. More generally, participants identified that women and families experiencing DFV struggle with long-term accommodation needs, the financial consequences of splitting families, and sometimes having to relocate from their home community.

Across the interviews and community fora, it was agreed that women and families experiencing DFV often experience a range of needs and have complicated care pathways that cross service delivery target issues, for example, mental health and drug and alcohol service needs. While appropriate services might be available, knowing about and navigating multiple services is challenging, as the next quote shows, “However, for a person to actually navigate through that system is an entirely different matter. It's like Pan's Labyrinth, the goal posts change all the time” (Interview, Health Sector Manager).

These factors make assisting women and families who have experienced DFV challenging in all time periods. However, specific challenges with service provision after-hours were encapsulated in the statement below from one interview participant who said:

You can’t say that you are able to meet everyone's needs after hours, and I would challenge anyone if they said they can meet all the needs after-hours, because you can’t. But you can put some things into place that will perhaps assist them until you’ve got time and a more reasonable hour to deal with it. (Interview, Accommodation Sector Frontline Worker)

The following sections summarize the main themes that emerged from both the stakeholder interviews and community fora. For the most part, themes were consistent across the two data collection methods. Where themes emerged specifically from only one of the data collection methods, this has been documented.

### Limited Available After-Hours Services

From the data collected, it became apparent that during after-hours periods, women and families experiencing DFV would most likely be assisted but that this assistance might look quite different to what they would receive during business hours. The participants indicated major issues impacting after-hours service delivery: a more limited range of services, the impact of distance and lack of transport, increased waiting times, generalist services working outside of their mandate, and inability to manage all elements of DFV cases where there are multiple or complex needs, for example, needs relating to disability, mental health issues, or drug and alcohol use. Although outlined here as separate issues, the reality is that these issues are interrelated. For example, available services may not be funded to assist with DFV, and appropriate referrals may not be located in the same town. This means that transport is difficult to arrange, in general and after-hours in particular, and that women and families may face long waiting times for assistance.

Participants in the interviews and community fora acknowledged that availability of services during after-hours (and in some instances in business hours too) is problematic in the small rural towns and regional centers of the Murrumbidgee region. Indeed, an interview participant spoke of their interaction with a woman–survivor of DFV in which the woman discussed the limited services available to her at night:

She said to me ‘Just come with me one night at nine o’clock and I’ll show you why I don’t do anything [about DFV]. In [community] there's only a service station open that's about a kilometer and a half out of town, and the hospital after nine o’clock at night, and the public toilets behind. That's all that's open. (Interview, Health Sector Manager)

For the most part, participants believed that some degree of after-hours services are available to women and families specifically through police, hospitals, crisis accommodation services, and telephone support. However, provision of after-hours services is not unproblematic in these service settings given that they are not all specifically designated for assisting DFV victim–survivors.

### Police and After-Hours Services

Depending on the community, the police station may or may not be a potential resource for women and families experiencing DFV. Some of the smaller target communities do not have 24-hour police stations, with after-hours services provided either by an on-call officer or by a station in an adjacent town. Access to assistance can be further compromised when available police officers are already out on a call. An interview participant highlighted this issue in their discussion of the realities of accessing police assistance after-hours in their local community.

Our police station shuts most nights ten o’clock or something like that, or even earlier, seven o’clock I think it is, so there's nowhere for them [DFV survivors] to go. They go down to the police station, and there's a phone to pick up and talk to somebody that's an hour away. That can be an issue. (Interview, Health Sector Manager)

A number of participants also noted that, while the police may be available as a first point of contact for women and families experiencing DFV, their job description and role, as well as availability of staff during after-hours, have a significant impact on what they are actually able to do for victim–survivors. In one of the community fora, a participant noted that “The only people they [DFV survivors] really see are the police who are taking details and moving on. They’re not there to hold their hand and help them in any way” (Community Forum 4).

This point was prefaced by discussion of the need for additional support and potentially counseling for DFV victim–survivors, particularly during after-hours. It was made in the context of a recognition that small communities are losing the ability to interact with clients and that personal relationships are needed to provide client-centered and trauma-informed services. A community forum participant touched on this when she mentioned the centralization of services.

All our services are being centralised, which is horrible, because they centralise it in a big place and the people in the big place have no concept of what happens out here … Our people who really need us aren’t going to ring and talk to a stranger. They’ll ring and talk to me, because I’ve got a relationship with them. (Community Forum 3)

An interview participant also picked up on the restricted ability of the police to provide for women's immediate needs during after-hours when she suggested that there might be potential for a new role in DFV support to assist the police.

It would be great if we had a non-government agency that was funded to ride with the police so that while the police are doing their job around safety, protection and arrest, that you did have another person there that was able to just provide a little bit more emotional support to the victim. (Interview, Police Sector Manager)

### Hospital Emergency Rooms

In some of the communities, participants acknowledged that the hospital was the only service open during after-hours; “Business hours is when all the services are available that are available. Hospital is really the only thing at night-time here” (Interview, DFV Service Sector Manager).

While hospital emergency departments were often the first port of call for women in the after-hours period, particularly if they had sustained physical injuries, hospitals were unable to provide an effective place for women to be in the aftermath of violence. In theory, hospitals can offer social admissions to women who do not have injuries requiring hospitalization but require short-term accommodation until other options are available during business hours. In reality, medical staff are reluctant to provide this option for a range of reasons that include concerns about staff and patient safety.

Most of our hospitals at night, you’ve got two people working in the hospitals, there's only two people on, so their security is just as important as anybody else's, so if somebody else was to come out, like the person that was causing the abuse was to turn up, police might be called, and that's the last thing these women want … it's a really, really tricky subject, that if our staff are in danger, they’d call the police. (Interview, Health Sector Manager)

In small communities, staffing levels in hospitals are low, and staff are therefore unable to conduct the necessary processes to ensure that DFV victim–survivors can be assessed and referred to the right services during after-hours. In one of the community forums, participants noted that only two nursing staff are available after-hours to manage more than 20 beds and any issues that come into the emergency room. They therefore do not have capacity to manage the referral or psychosocial needs of DFV victim–survivors who may use the hospital as their first option after-hours.

[there is a perception that] If the police brought them [victim] to the hospital and they’re injured, then the hospital could organise all this stuff [DFV Services]. They can’t. There's no way in the world that that limited staff can cope with the physical injuries and do any kind of organisation of DV services. (Community Forum 3)

### Accommodation

Many discussions in the interviews and community fora focused on challenges with the provision of accommodation for women and families experiencing DFV. Indeed, although this issue was discussed as specifically problematic in the after-hours crisis space, it was acknowledged as a barrier for assisting rural and regional women with DFV more generally.

It's almost impossible for women and children who wish to relocate, you know, from their – their dwelling where the violence has occurred into their own dwellings. It's virtually almost impossible to find safe, secure alternate type housing. (Interview, Accommodation Sector Frontline Worker)

Across the region, several different options exist to provide accommodation to women; however, all participants acknowledged that during after-hours, it was most likely to be crisis accommodation that was required and that this complicated service delivery. Options available for after-hours accommodation include crisis accommodation and access to local hotels using a voucher system if refuge accommodation is not available or doesn’t exist locally.

Several challenges were identified with all forms of accommodation. For crisis accommodation specifically, participants noted that accommodation targeted for DFV was being defunded and was less available than previously. They also discussed the challenges of finding accommodation for women with children (particularly male and over 10 years) given that DFV-specific accommodation is frequently designated as “women-only space” and would therefore not allow older male children to remain with their mothers. Staffing of crisis accommodation was specifically difficult in the after-hours space because most did not operate on a 24/7 basis and were therefore only able to process admissions during business hours or shortly thereafter. Demarcations in funding have narrowed the characteristics of clients able to access some accommodation, making them unavailable for DFV victim–survivors and therefore reducing the pool of accessible accommodation for women and families fleeing violence.

Once upon a time you could ring in the middle of the night and get somebody who was in the service who would do an assessment for you. Nine times out of ten if they had a room they would take that woman and their children. Now there's no after-hours service so therefore you’ve got to ring Link2Home. If they can’t find you somebody you can have a woman sitting in the police station at 3:00 in the morning. (Interview, Police Sector Manager)

An exception to business hour crisis accommodation, and a service that was discussed in almost all data collection activities, is the Griffith Links for Women crisis accommodation that is the only service in the region to offer 24/7 staffed access to accommodation. The fact that Links for Women is funded to provide care 24/7 increased accessibility, with the service having the ability to conduct assessments and accommodate women during after-hours and with limited delays. This has created a large demand for their services, with referrals being made from other areas of NSW and from out of state, a demand that has outstripped their available resources.

Link2Home and other organizations operate by pairing women with motels in their local area that have rooms available for DFV victim–survivors. In reality though, availability in motels is often limited. Reception is not staffed 24/7, and therefore, during after-hours, there is no one available to contact to arrange for women to move into the accommodation. Many motels are not willing to keep specific rooms open and available during busy times.

If you’ve got a domestic violence victim at ten o’clock at night who needs accommodation you ring your local service who doesn’t have an after-hours support worker on board. You would have an on-call worker who would say to you, “I’m the on-call worker. We’re unable to take admissions at night. You will have to phone back in the morning.” You then phone Link2Home which is the after-hours homeless accommodation line. They might say, “Yes. We can help you,” but they may ring around the motels in this area and at ten o’clock at night very few motels will even take anybody that's coming – that's travelling through town rather than someone who's a DV victim. (Interview, Health Sector Manager)

Several participants reported that motels were increasingly reluctant to participate in the program after negative experiences with perpetrators tracking down partners, perpetrating more violence, and creating a disturbance in the motels that form the backbone of people's businesses.

While concerns about privacy have been cited as issues for women managing DFV in small communities, there was a sense from some of the interview and forum participants that such communities might actually provide additional resources in the DFV space, particularly given the emphasis that participants placed on the importance of relationships.

[Small communities] can be a safety factor because that close friend, the family ties, that type of thing, often, I guess to some extent, often negate the need for an immediate placement for someone. And I have worked in the homelessness sector, and if we could place anyone with family, whilst we acknowledge that it's not a long term …, it's not permanent, it's not a great solution …, it is a healthier solution for them than planting them in a refuge. Irrespective of what anyone likes to think, a refuge is just another form of an institution, and we have people … in the long term, they become institutionalised like they do in many other institutions. (Community Forum 2)

### Telephone Support

For the most part, participants agreed that provision of support, information, and some level of counseling is available in most communities in the Murrumbidgee region. This is largely facilitated by state-wide or national helplines specifically targeting the needs of women experiencing DFV. The perception among service providers is that this is largely for the provision of information and does less in terms of counseling: “Then there are all the other hotlines 13 RESPECT, and things like that, that can provide some information, but it's not really support.” (Interview, Health Sector Frontline Worker)

Some participants questioned the service that telephone support can offer, drawing on a common theme about interpersonal relationships being a necessary component of the DFV response that cannot be provided through phone lines and/or telehealth. A forum participant made this point when she said, “‘Ring the DV line, there's 24/7 counseling services available.’ It's not always appropriate, especially when you’re applying trauma-informed care and you can see them needing something, an immediate need” (Community Forum 4).

A number of participants talked about how control of mobile phone access has become a common facet of DFV. While telephone support may be available during after-hours, there were many concerns expressed about the practicalities of actually accessing such support in instances where public phones are increasingly being decommissioned and where a perpetrator may be controlling access to a mobile phone or may have stolen or damaged it. During after-hours, locations in which women can have safe and confidential conversations about DFV are limited, even if women do have access to a mobile telephone.

We talked about where they would go to. For a start, there's no public telephones in the community, so if they haven’t got their mobile phone … Even where they’d go to talk to somebody on an access line. You’d sit on a park bench in the dark down in the main street or try and get to the service station out there and even if there's that line, where do you find an area that you’ve got privacy to talk to somebody on an access line or something like that? (Interview, Health Sector Manager)

Finally, participants discussed that after-hours phone numbers change frequently and that resources are not updated. Information about telephone lines that is accessible to women during after-hours (such as might be accessed through mobile phones) cannot therefore always be assumed to be accurate and useful. Indeed, in the preparation of resources for community fora, we found that a large number of support services advertised online were not available anymore or that the telephone numbers were out of date.

### Transport, Long Distances, and Boundaries

Across all data collection mechanisms, and particularly for smaller towns, transportation to access services or escape violence in an emergency was identified as a very real gap for both women and families and the services themselves.

A lot of the people actually live in-between in small villages that are, say, five hundred to a thousand, or two thousand people. A lot of organisations will either not travel there or travel there so sparsely that it is ineffective for the person. (Interview, Health Sector Frontline Worker)

The ability of organizations to use their own transportation to visit women and families at their homes or to provide victim–survivors with transport to services is limited by concerns about safety of their workforce, safety for clients, and client focus areas determined by funding. A specific issue was safe transportation of children in car seats. Some service providers spoke about not being able to assist women because they do not have car seats available for the transportation of children. In some instances, they mentioned being aware that other services have the necessary car seats but are either not funded for DFV or for after-hours services and are therefore inaccessible at the times that women and families need them. In these (and other instances), service providers reported a willingness to work around the rules in order to ensure that they assist women and families in need.

Other services discussed using whatever transport might be available to move clients to referrals during after-hours.

She may have family there that they are able to transport her or we’re able to … and [local town] is probably not a good [example] because they don’t have a taxi there, right, so … but the police might transport her to a family member, then we’ll pick her up the next day, and, you know, if it's [local town], we might meet them halfway, so there's ways around it. (Interview, DFV Sector Frontline Worker)

Specifically in smaller communities, there was discussion of the lack of safe community or public transport. In these communities, transport options are so limited that they become an identifiable location for perpetrators who may want to find their victims: “She's going to be on that bus at 9 o’clock in the morning. Unless these creative solutions exist” (Community Forum 3).

Service providers spoke of therefore hiding women and transporting them to the closest town where they could catch the train/bus without being seen at known local transport hubs. Finally, in the context of many DFV victim–survivors needing to access a range of services, participants spoke of the challenges across large distances, of managing different service areas for different types of services.

Our difficulty is that we’re in the corner of everything. So, we’re on the far corner of Lachlan Shire. We’re in Murrumbidgee for health. We’re in Parkes for police. So, everyone is working out of different areas. DoCS [Department of Community Services] – Brighter Futures is from Orange. Where we think it would be much better for us to get a Brighter Futures service from Griffith, because it's an hour and a half away, not four hours. But the way it's been divided out, and this is the problem. We’re crossing across boundaries all of the time. (Community Forum 3)

In combination with the challenges that participants noted with regard to availability of transport, the need to access services in a number of different directions impinged on the effective response to DFV, at all times, but specifically during after-hours when already scarce public transport is entirely unavailable.

### Long Waiting Times

Across many of the discussions, there was an acknowledgement that while services were generally available in some form, the after-hours time period was particularly problematic for the speed in which women might access those services . Numerous participants discussed instances where women were forced to wait for long periods of time while referral services were located, counseling staff arranged in smaller towns, or crisis accommodation secured. In many instances, participants discussed the fact that women became impatient with waiting and often returned to their homes where the violence had been initially perpetrated. This was particularly problematic for services available after-hours, but which are not specialized DFV services. In these instances, staff often do not have time to address immediate needs such as assessment and referral to services.

We’ve had a DV victim who is at the hospital. Since the police took them there the violence workers at the hospital couldn’t get this lady accommodation. They can keep them overnight if they absolutely have to, but this woman got sick of waiting for an accommodation option and she walked out of the hospital at four o’clock in the morning and went back home to where she’d been beaten. (Interview, Health Sector Manager)

### Ability of Services to Meet DFV Needs

Across most interviews and community fora, there was discussion of the inability of existing services to meet the DFV needs of rural communities, during after-hours and in usual business hours. Numerous providers discussed challenges and complexities in the service delivery space and how this impacted on their ability to assist DFV clients. Two major issues were identified: First, increased funding pressure means that service organizations’ mandates are becoming more focused on individuals who meet specific criteria. For example, an organization might be funded to assist women with children in a particular age range, rather than women more generally. Services are therefore limited in terms of the response that they can offer at critical times if the client does not match their criteria. This is particularly relevant during after-hours when there are already limited service options available, and this is compounded by eligibility barriers. Second, within services, there is a reduced ability to assist DFV clients who have complicated and multiple needs. In particular, this means that women and families with more complex needs either cannot access the services they need or are forced to access services across multiple organizations, often located in several distant locations. However, numerous participants pointed out that despite narrow organizational mandates, they continue to assist women experiencing DFV, even when women do not meet the criteria for accessing their particular service.

We are often helping people that we’re not funded to help. (Interview, DFV Service Sector Manager)

Across the board, the main sentiment in these discussions was a willingness to bend the rules in the interests of assisting women and families, although this may contribute to increased workload for which the organization cannot account.

There are restrictions on all of us; around what we can and can't do and we really need to look after each other in supporting that agenda. The other thing is, is when we do a more coordinated response to families or individuals, then we also reduce that level of worker safety components. Because, you don’t have to visit alone, maybe we could do something jointly. If we’ve got a visiting service, maybe you can use our office to do that so you’re not in the home. How do we reduce those remote, isolated issues that come with where we live and how we do business? (Community Forum 3)

As can be seen from the quote above, service providers are willing to adapt their operations in this manner—indeed, would welcome greater opportunities to assist one another within (or outside) the confines of their specific funded criteria.

With regard to clients with complex needs, such as drug and alcohol dependence or mental health issues, provision of DFV services was noted as becoming particularly challenging. In terms of after-hours services, participants spoke specifically about accommodation not being readily available for women who might reveal drug and alcohol or mental health issues during assessment. DFV-specific services lack the skills and resources to effectively manage such women, and concerns about staff and other residents’ safety are given precedence during decision making. Participants recognized that this is often the reality of working in the DFV field and that the system should accommodate this reality.

Participants raised concerns about service systems not being sufficiently client centered. A forum participant spoke of the need to meet target numbers before services are prepared to travel to her small community to provide services, noting:

A very common theme we hear when it comes to particularly, say mental health services, working with men, counselling services, et cetera is “when the need arises.” What does the need to look like? I have to say as a community person and a previous service provider, there is nothing more offensive than being told, “we’ll come when the need arises, as long as it fits our need and not community need.” That's something that I guess we talk about a lot, is when we design service provision, let's design a service provision with community so that it is fit for purpose, rather than fit for service. (Community Forum 3)

## Discussion and Conclusions

This research aimed to understand the service landscape in the after-hours period in target communities in the Murrumbidgee region and to identify service gaps and barriers. We found a perception that there is limited, successful service provision in the after-hours period in our target communities, yet there is also perceived room for development and improvement as the needs of women and their families are not being sufficiently met. We considered our findings through the lens of Kennedy et al.'s (2012) conceptual model of help attainment for women experiencing violence. Specifically, we focused on the experience of intervention, rather than the mental health outcomes that are included in the model. Kennedy et al.'s (2012) model identifies women's experience of help seeking in terms of their social location and situational context (in this case, often low socioeconomic, small rural communities and in the after-hours space) and explores attainment of assistance, rather than just access. This was important for our analysis as, although some after-hours services exist, they are not always timely or accessible for women and their families, and do not work equally well for all community members, or for all communities. Although it may be tempting to view online and telephone resources as sufficient during after-hours in nonmetropolitan communities, technology is not a straightforward or unproblematic panacea for the challenges of delivering services. It may be difficult for a person leaving DFV to access technology such as a mobile phone because it has been taken from them as part of the perpetration of violence, or they have left a situation quickly, without bringing their device. Additionally, due to cost and connectivity challenges, rural Australians experience a “digital divide” meaning their access to the internet and phone connectivity is significantly less than that of Australians in metropolitan and regional areas (George & Harris, 2014). Second, technology can provide a site or target for DFV (Bailey et al., 2007). A Victorian study identified that it was common for survivors to experience technology-facilitated abuse and stalking while using information and communication technologies (George & Harris, 2014).

Participants in this study were vocal about the scarcity of crisis accommodation during after-hours, echoing findings from a report examining progress since the *Royal Commission into Family Violence* and other research conducted in this local area (Schineau & Darley-Bentley, 2016; Victoria State Government, 2019). Reforms in the 2014 NSW Specialist Homelessness Services (SHS) altered the process in which DFV services were delivered and funded in NSW. A key element of change included funding some services that had traditionally focused on homelessness to provide support to victim–survivors of DFV as evidence emerged of the growing problem of homelessness among women in domestic violence situations (Zufferey, 2008). The majority of DFV services in NSW are now part of the SHS sector (Domestic Violence NSW, 2017), and demand frequently overwhelms available resources, despite efforts to reduce the likelihood of homelessness due to DFV by relying on partnerships with homelessness accommodation services, motels, and estate agencies to support women experiencing DFV (Chung et al., 2000; Wendt et al., 2015).

During the data collection, two clear issues emerged from participants about the after-hours service landscape; first, that many of the gaps identified by participants in DFV services were not limited to only after-hours services. Participants found it challenging to keep their comments focused on discussing after-hours needs rather than needs for DFV victim–survivors more generally. Second, participants tended to conflate after-hours services with crisis responses to DFV events, yet there may be after-hours requirements that are not associated with an immediate crisis. This distinction did not emerge in our data.

It is important to highlight that the services and individuals that participated in this research displayed incredible tenacity, flexibility, creativity, and value-driven work that has helped women and families find safety from DFV, despite the barriers to service delivery that they must overcome. Some after-hours services were provided, sometimes as a direct result of the lengths that workers have gone to, at times beyond their policy, service, and funding boundaries. Nevertheless, major challenges were identified across the target areas: a lack of information and a lack of access to services.

The lack of information became apparent when every participant commented that they had learned about a service during data collection that they were not previously aware of, despite working in a related service. While our data and analysis in this study were focused on the impact of service complexity on victim–survivors, the provision of appropriate services requires that providers are able to assist victim–survivors in navigating the system. Where the system is constructed of a multitude of services with specific and narrow eligibility criteria, across geographical boundaries, and in a context of rapid funding change, the ability of providers to direct women to appropriate services is greatly challenged.

There were several context-specific features of available interventions that prevented women from attaining care and support in both the after-hours and business hours periods; we focus here on after-hours issues. The first barriers included a lack of public, or other transport options, large distances between communities, limited services available in the after-hours period (such as accessible, safe housing), access to the internet, and conflicting service boundaries, resulting in access complications for different DFV generalist and specialist services. Census data show that fewer than 50% of households in each of the target communities have more than one vehicle (Australian Bureau of Statistics, 2021). In the context of leaving a violent situation or relationship, the ability to access public or private-paid transport (such as a taxi) may be the only way to find safety. Lack of transport options was noted in every community, but the most remote community had a particularly pronounced need. Similarly, the target communities included a higher than state average number of households with no internet access, including via mobile telephone data, likely limiting telephone helpline access and the ability to access information about services during after-hours (Australian Bureau of Statistics, 2021). While crisis accommodation was available in theory, in practice, it was perceived to be frequently inaccessible. Having no safe space for extended time periods because of intake processes places women and families in danger.

Once a victim–survivor was able to identify a service (or find support to do so), then additional barriers to actually attaining assistance emerged including service criteria that exclude some community members from accessing services, services that are not adequately equipped to support people with complex needs, and service policies that create access barriers, for example, services that are not allowed to transport service users. Where service policies sometimes prevented staff from offering services at all (because they are out of funding scope) or taking necessary steps to ensure the safety of women and children (e.g., by providing transport), staff were faced with either disservice to women, children, their employer, or themselves. Available services in the after-hours period might be accessible, for example, emergency departments, but their ability to offer actual assistance is often constrained.

The provision of services for women and their families experiencing DFV is challenging and is made more so when operating in the after-hours space and outside of comparatively well-resourced metropolitan areas. Although it has long been acknowledged that distance makes for exigent service delivery (Owen & Carrington, 2015), our work in this particular area notes that the structural and administrative parameters directing DFV services create additional complications for effective service delivery during after-hours in regional, rural, and remote communities. When viewed through the lens of Kennedy et al.'s (2012) model, it is clear that the intersection of social location (rural), resources (availability of specific services), and situational context (the after-hours space) significantly impact women's opportunities to attain help and support when needed. While one of our participants noted that “you can’t meet everyone's needs after-hours,” we should be working towards a better suite of options for women and their families whose experiences of DFV fall outside office hours and are located in the more marginal geographic spaces of Australia.

### Limitations

The limitation of this project are that we were not able to identify the particular perspectives of victim–survivors of DFV in the target communities, despite our original intention to do so.^1^ The views of victim–survivors and their experiences accessing services during after-hours are an important gap that ought to be addressed by future research. While the community fora were intended to accommodate victim–survivor's viewpoints, most attendees were service providers. Moreover, uptake of invitations to the community fora was limited, and the small number of participants across multiple communities is a limitation of the study. Interview participants do not include individuals from the legal sector, Local Health Advisory Committees or Aboriginal Medical Services, as potential participants in these sectors declined to be interviewed when approached. Following the literature that documents particular barriers for Indigenous survivors in Australia (such as Maher & Segrave, 2018; Murray et al., 2019), we hoped to highlight the particular perspectives of Indigenous survivors and service providers. However, we did not collect sufficient data on these particular experiences. This is an important focus for future research.

## Notes

1. In Australia and internationally, there is no agreement about terminology used to describe gender-based violence that occurs in family contexts and more commonly effects women. In this research, we use the term “domestic and family violence” and then DFV to be inclusive of the variety of forms of violence and understandings of family in different cultural contexts. However, we have retained the chosen term used by participants in the findings section to accurately convey the data.

2. The research sites were classified as either inner regional, outer regional, or remote communities in NSW, Australia. This research uses the definition provided by the Australian Bureau of Statistics. Remoteness is measured by road distance to urban centers. For a comprehensive description of this measurement, please see https://www.abs.gov.au/ausstats/abs@.nsf/Latestproducts/1270.0.55.005Main%20Features15July%202016?opendocument&tabname = Summary&prodno = 1270.0.55.005&issue = July%202016&num=&view = .

3. The research was approved by the Greater Western NSW Human Research Ethics Committee (2019/ETH03957) and the Aboriginal Health and Medical Research Council NSW (1516/19).

4. The Project Steering Committee was convened of individuals working in a range of sectors associated with DFV in the target communities.

5. Some participants acted in both management and frontline worker roles due to the service size or resources.
